# Human immunodeficiency virus type 1 impairs sumoylation

**DOI:** 10.26508/lsa.202101103

**Published:** 2022-02-18

**Authors:** Bilgül Mete, Emre Pekbilir, Bilge Nur Bilge, Panagiota Georgiadou, Elif Çelik, Tolga Sutlu, Fehmi Tabak, Umut Sahin

**Affiliations:** 1 Department of Infectious Diseases and Clinical Microbiology, Istanbul University-Cerrahpasa, Cerrahpasa School of Medicine, Istanbul, Turkey; 2 Department of Molecular Biology and Genetics, Bogazici University, Center for Life Sciences and Technologies, Istanbul, Turkey; 3 Department of Medical Biology, Istanbul University-Cerrahpasa, Cerrahpasa School of Medicine, Istanbul, Turkey

## Abstract

During infection, the human immunodeficiency virus type 1 (HIV-1) manipulates host cell mechanisms to its advantage, thereby controlling its replication or latency, and evading immune responses. Sumoylation is an essential post-translational modification that controls vital cellular activities including proliferation, stemness, or anti-viral immunity. SUMO peptides oppose pathogen replication and mediate interferon-dependent anti-viral activities. In turn, several viruses and bacteria attack sumoylation to disarm host immune responses. Here, we show that HIV-1 impairs cellular sumoylation and targets the host SUMO E1–activating enzyme. HIV-1 expression in cultured HEK293 cells or in CD4^+^ Jurkat T lymphocytes diminishes sumoylation by both SUMO paralogs, SUMO1 and SUMO2/3. HIV-1 causes a sharp and specific decline in UBA2 protein levels, a subunit of the heterodimeric SUMO E1 enzyme, which likely serves to reduce the efficiency of global protein sumoylation. Furthermore, HIV-1–infected individuals display a significant reduction in total leukocyte sumoylation that is uncoupled from HIV-induced cytopenia. Because sumoylation is vital for immune function, T-cell expansion and activity, loss of sumoylation during HIV disease may contribute to immune system deterioration in patients.

## Abstract (precis)

The HIV type 1 dampens host cell sumoylation in vitro and reduces the expression of UBA2 protein, a subunit of the SUMO E1–activating enzyme. In vivo, infection in patients is associated with diminished global leukocyte sumoylation activity.

## Introduction

The human immunodeficiency virus type 1 (HIV-1) replicates primarily in CD4^+^ T-helper lymphocytes of the immune system, and to a limited extent in dendritic cells and macrophages (1). Along the course of untreated HIV-1 infection, as the viral load progressively increases, the immune function collapses due to the depletion of circulating CD4^+^ T-cells (1, 2, 3). Clinical manifestations of AIDS include various opportunistic infections and specific cancers, and often present once the number of CD4^+^ T-cells falls below 200 cells/mm^3^ (4). During infection, HIV-1 interfaces with multiple cellular pathways and proteins, hijacking some to its advantage for efficient replication or for latency control, and targeting others to disarm host immune responses (5, 6, 7, 8). Among these, post-translational modifications (PTMs) constitute an emerging class of interactors that can either promote or restrict viral replication (9, 10, 11, 12). For example, the spike proteins gp120/gp41 are subject to glycosylation before their cleavage from the gp160 precursor (Env), which is necessary for further maturation and assembly (13, 14, 15). Phosphorylation of HIV-1 reverse transcriptase by Cdk2 or casein kinase II (CK2) can stimulate its distinct functions such as RNA-dependent polymerization or ribonuclease H activity (9, 16, 17, 18, 19). HIV-1 integrase is a key drug target that plays a central role in the incorporation of viral DNA into the host genome. This critical enzyme undergoes multiple PTMs including phosphorylation, acetylation, ubiquitination, and sumoylation (20). The biochemical consequences of these modifications are diverse, some modulating the affinity of integrase to DNA, whereas others regulate the enzyme’s stability.

Sumoylation is an essential PTM with crucial roles in eukaryotic cells (21). Sumoylation involves reversible modification of substrate proteins on target lysine residues by covalent conjugation of a small, 18 kD, ubiquitin-like peptide called small ubiquitin-like modifier (SUMO) (21, 22). While SUMO shares only 18% sequence identity with ubiquitin, the overall structural similarity between these two peptides is remarkable (23). Similar to the ubiquitylation cascade, SUMO attachment occurs through the coordinated efforts of a set of enzymes, including the E1 (activating) and the E2 (conjugating) enzymes, as well as a limited set of SUMO E3 ligases (21, 22, 24). In the first step of this conjugation cascade, the mature SUMO peptide is activated in an ATP-dependent manner by the SAE1/UBA2 heterodimer (E1 enzyme). After activation, SUMO is transferred onto UBC9, the unique E2 enzyme of the pathway, which is capable of directly catalyzing the attachment of SUMO to target lysine residues that reside in specific sumoylation motifs on substrate proteins. Often, these motifs are comprised of ψKxD/E where ψ denotes a large hydrophobic residue and x any residue, however, extended or even reverse consensus motifs have been reported (21, 22).

At the biochemical level, conjugation of SUMO can alter various properties of target proteins, including their enzymatic activity, subcellular localization, stability, or interactor profile (25). Consequently, a plethora of cellular functions may be modulated by sumoylation, including gene expression and transcription, signal transduction, cell division and proliferation, apoptosis, senescence, stress response, and stem cell renewal (26, 27). The essential roles played in the cell by sumoylation have been documented in knockout studies in which abrogation of UBC9 was shown to cause lethality in yeast and mice, resulting from gross defects in cell division, nuclear architecture, and chromatin integrity (28, 29, 30).

The human genome encodes four SUMO paralogs: SUMO1, 2, 3, and 4. SUMO2 and SUMO3 share 97% similarity and are collectively referred to as SUMO2/3 (21). SUMO2/3 carries an internal sumoylation consensus motif, as well as other non-consensus sumoylation sites, allowing the formation of long SUMO chains with distinct branching patterns, as also observed with ubiquitin (31). SUMO1 lacks such an internal consensus motif and thus usually participates in these chains as a terminator cap. Nevertheless, recent studies indicate that under certain stress conditions SUMO1 can also be sumoylated on non-consensus lysines, hinting at the existence of complex mechanisms regulating the poly-SUMO chain topology (32, 33). SUMO-targeted ubiquitin ligases (STUbLs) are a specific class of ubiquitin E3 ligases that preferentially recognize poly-SUMO chains and catalyze the addition of ubiquitin either to the growing SUMO chain or to a neighboring lysine residue on substrate proteins (34, 35). This way, poly-SUMO chains can serve as a platform to initiate substrate ubiquitination and proteasomal degradation (34, 35). Because sumoylated substrates can undergo both proteolytic and non-proteolytic ubiquitination, signaling through poly-SUMO or hybrid SUMO-ubiquitin chains can control a spectrum of cellular responses, especially under stress conditions (21). Whereas a limited pool of unconjugated SUMO1 is maintained in vivo, SUMO2/3 is abundantly expressed and rapidly mobilized to be conjugated to target substrates in response to DNA or protein damaging stress, such as heat shock or oxidation (36, 37, 38). In line with this observation, SUMO2/3 was found to be essential for cells to survive through hyperthermic shock (37). These and numerous other studies therefore indicate that modification by SUMO1 and SUMO2/3 may have distinct physiological outcomes, the latter particularly emerging as a crucial mediator of cellular and organismal responses to physiological or environmental stress conditions, including infection (21).

SUMOs indeed play essential roles in anti-microbial defense and also in the immune system (21, 39, 40, 41). In vitro in cultured cells, depletion of SUMO proteins favors pathogen replication, whereas SUMO overexpression opposes replication of viruses and bacteria (42, 43). For instance, SUMO proteins are now emerging as important mediators of the interferon response (43). Interferon stimulation increases the expression of SUMO proteins in a microRNA-controlled manner, and abrogation of sumoylation blunts interferons’ antiviral activities in a number of viral infections in vitro, including HIV-1 (43). Sumoylation is also intricately linked to other signaling pathways that are critical for the immune system, such as NF-κB and Toll signaling (39, 44, 45). At the physiological level, sumoylation regulates T-cell expansion and function, as well as inflammatory responses (45).

Remarkably, during infection, several viral and bacterial proteins down-regulate global sumoylation to disarm host immune responses (45). Human Herpes Simplex Virus 1 (HSV1) protein ICP0 is among the best characterized of such proteins. ICP0 is a STUbL that targets a subset of sumoylated host proteins for proteasomal destruction, including those with anti-viral activities such as PML and SP100 proteins (46, 47, 48, 49). Similarly, during infection by *Listeria monocytogenes*, the bacterial virulence factor listeriolysin O (LLO) induces proteasome-independent degradation of host UBC9, thereby decreasing sumoylation and dampening the innate immune response (42). A number of HIV-1 proteins, including the integrase enzyme, are known to be sumoylated (10, 20, 50, 51). However, little is known about the reciprocal interactions between HIV-1 and host sumoylation, particularly when it comes to the potentially subversive effects of the former on the latter. In this study, we demonstrate that HIV-1 expression and replication in cultured HEK293 or HeLa cells, or in CD4^+^ Jurkat T lymphocytes can cause a dramatic reduction in host cell sumoylation by targeting the UBA2 subunit of the SUMO E1–activating enzyme. We also show that HIV-1 infection in patients is associated with a global demise in total leukocyte sumoylation. Whereas further research is necessary to elucidate the mechanistic basis of sumoylation loss in leukocytes in vivo, our data indicate that this is uncoupled from CD4^+^ cell depletion, but rather directly linked to uncontrolled viral replication.

## Results

### HIV-1 impairs cellular sumoylation in vitro

**Figure S1.**
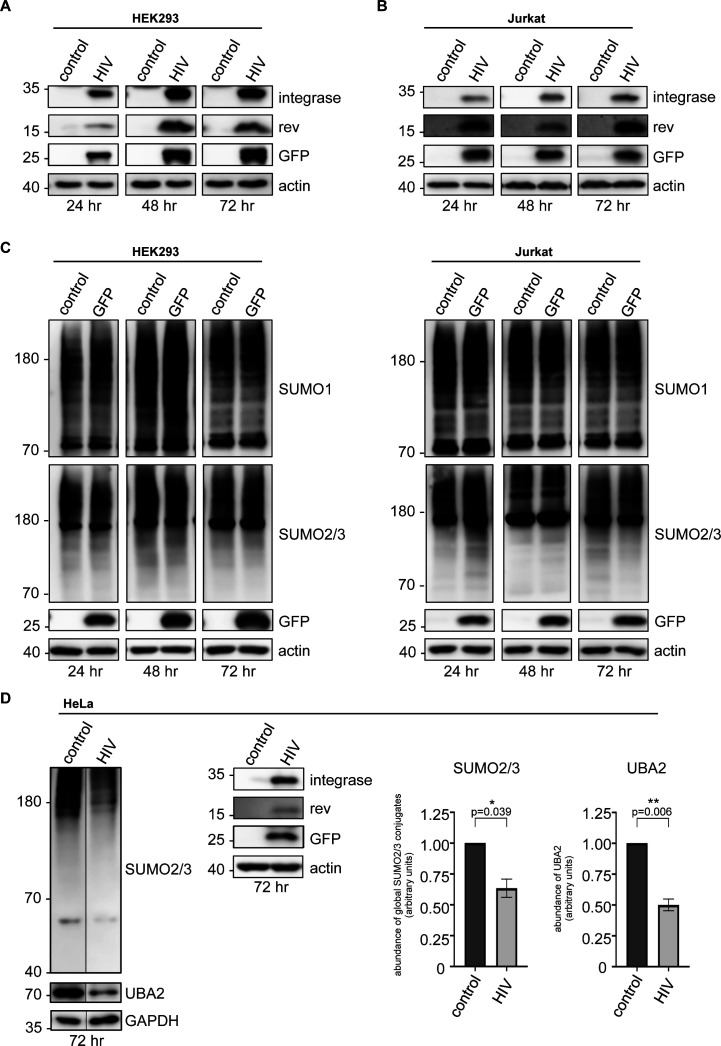
Viral protein expression in HEK293 or Jurkat cells, and HIV-1–induced down-regulation of cellular sumoylation. **(A, B)** Assessment of EGFP, HIV-1 Rev, and HIV-1 integrase expression by Western blot in HEK293 cells (A), or in CD4^+^ Jurkat T lymphocytes (B), transfected or electroporated with the lentiviral vector pfNL43-dE-EGFP encoding HIV-1. The EGFP cassette was inserted within the HIV-1 *env* gene. **(C)** HEK293 cells or CD4^+^ Jurkat T lymphocytes were transfected or electroporated with a vector encoding EGFP only. Global SUMO1 and SUMO2/3 conjugates were analyzed by Western blot. **(D)** HeLa cells were transfected with the lentiviral vector pfNL43-dE-EGFP encoding HIV-1, and lysed 72 h post-transfection to assess global sumoylation levels, as well as UBA2, EGFP, HIV-1 Rev, and HIV-1 integrase expression by Western blot. Densitometric quantifications of SUMO and UBA2 signals were normalized to GAPDH expression (which serves as a loading control). Data are presented as mean ± SEM (n = 3), *P*-values were calculated using *t* test assuming unequal variances. Control: cells transfected with an empty vector backbone devoid of HIV-1 genes, HIV: cells expressing the HIV-1 genome.

To simulate HIV-1 infection in cell culture, we transfected HEK293 cells or electroporated Jurkat cells, an immortalized line of human CD4^+^ T-cell lymphocytes, with a lentiviral vector (pfNL43-dE-EGFP) encoding the HIV-1 genome whose expression is driven by long terminal repeats. This virus is capable of reverse transcription, integration, replication, and expression of all viral genes (including *gag*, *pol*, *tat*, *rev*, *vif*, *vpr*, *vpu*, and *nef*) encoding the structural, accessory, and regulatory HIV-1 proteins in transfected cells, but incapable of forming viral particles due to the impairment of envelope protein production by an EGFP cassette that is inserted within the *env* gene. We verified the transfection (or electroporation) efficiency by following EGFP expression by Western blot (Fig S1A and B). In addition, in both HEK293 and Jurkat cells, Western blot analyses confirmed the expression of viral integrase and Rev proteins (Fig S1A and B).

**Figure 1.**
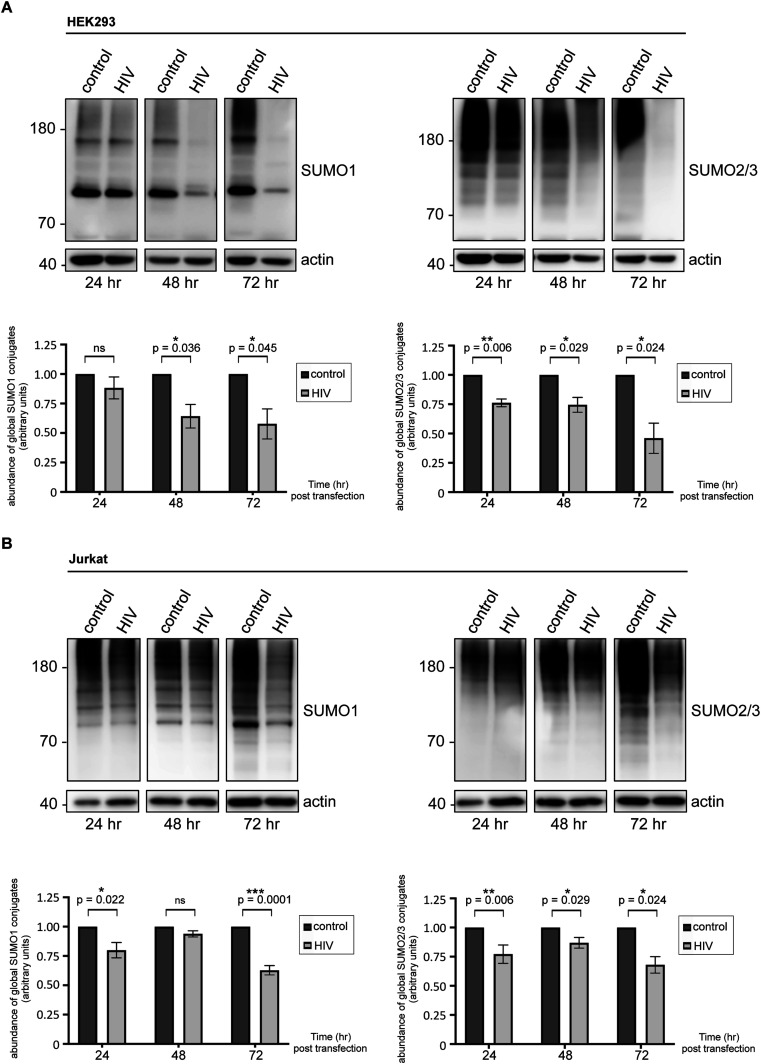
HIV-1 diminishes the abundance of cellular SUMO conjugates. **(A, B)** A lentiviral vector (pfNL43-dE-EGFP) encoding HIV-1 (containing all nine viral genes: *gag*, *pol*, *tat*, *rev*, *vif*, *vpr*, *vpu*, *nef*, and *env*) was transfected into HEK293 cells (A) or electroporated to CD4^+^ Jurkat T lymphocytes (B), allowing reverse transcription and integration, followed by viral protein expression in cells. Neither the envelope protein nor any viral particles are produced because of the insertion of an EGFP cassette within the *env* gene. Expression levels of EGFP and two viral proteins, Rev and integrase, are shown in Fig S1A and B. This system allows in vitro simulation of HIV-1 infection in a safe, reproducible and quantifiable manner. Cells were lysed at indicated times post-transfection, and global sumoylation levels were assessed by Western blot, using human anti-SUMO1 and anti-SUMO2/3 antibodies. Representative blots are shown. Densitometric quantifications of SUMO signals were normalized to actin expression (which serves as a loading control). Data are presented as mean ± SEM (n = 4 for HEK293 cells, n = 7 for Jurkat cells), asterisks denote statistical significance (*P*-values were calculated using *t* test assuming unequal variances, ns, not significant). Control: cells transfected with an empty vector backbone devoid of HIV-1 genes, HIV, cells expressing the HIV-1 genome.

Remarkably, 48 h post-transfection with the HIV-1 genome, the level of SUMO1-conjugated proteins, as well as of those conjugated with SUMO2/3 declined significantly in HEK293 cells, compared with the cells transfected with a control vector devoid of HIV-1 genes (Fig 1A). Cellular sumoylation by both SUMO1 and SUMO2/3 continued to diminish sharply over the course of 72 h. Critically, we also observed a significant decrease in both SUMO1- and SUMO2/3-conjugated proteins in HIV-1–expressing Jurkat cells (Fig 1B). Importantly, we did not observe any reduction in cellular sumoylation upon transfection or electroporation of these two cell lines with a vector encoding EGFP only (Fig S1C). Similar results were also obtained in HeLa cells expressing the HIV-1 genome (Fig S1D).

Collectively, our data indicate that HIV-1 expression in multiple cell lines, that entails its replication, integration and viral protein production, can precipitate a robust decrease in cell-wide sumoylation, implying that the virus may target this PTM during infection.

### HIV-1 induces a specific loss of UBA2 protein and interferes with SUMOs’ conjugation

**Figure 2.**
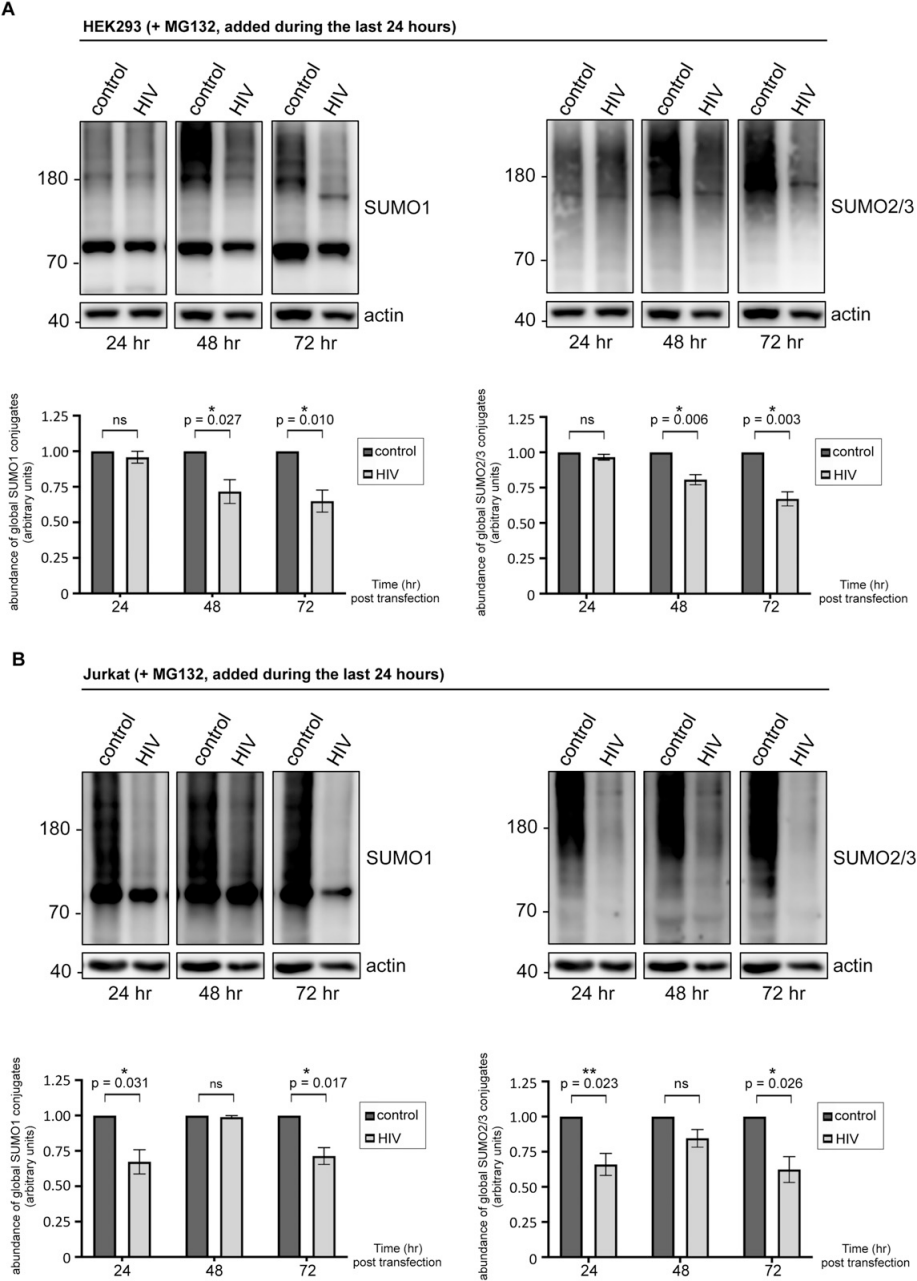
HIV-1 induces proteasome-independent loss of cellular SUMO conjugates. **(A, B)** HEK293 cells (A) or CD4^+^ Jurkat T lymphocytes (B) were transfected or electroporated with the lentiviral vector pfNL43-dE-EGFP encoding the HIV-1 genome as described above, then treated for 24 h with 3 μM MG132, a proteasome inhibitor drug, before lysis (at indicated times post-transfection/electroporation). Global SUMO1 and SUMO2/3 conjugates were analyzed by Western blot. Densitometric quantifications of SUMO signals were normalized to actin expression (which serves as a loading control). Data are presented as mean ± SEM (n = 5 for HEK293 cells, n = 4 for Jurkat cells), asterisks denote statistical significance (*P*-values were calculated using *t* test assuming unequal variances, ns: not significant). Control: cells transfected with an empty vector backbone devoid of HIV-1 genes, HIV: cells expressing the HIV-1 genome. Ubiquitin blots are shown in Fig S2.

**Figure S2.**
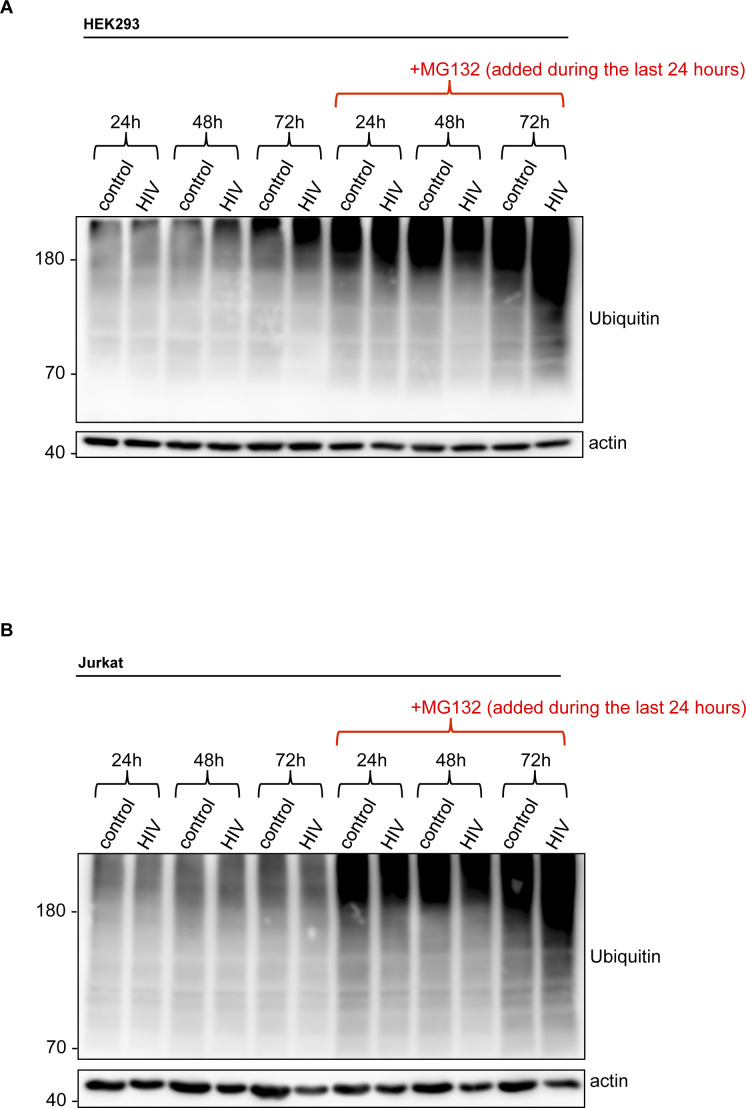
Ubiquitin conjugates in MG132-treated cells. **(A, B)** Western blot analysis of global ubiquitin conjugates of HEK293 cells (A) and CD4^+^ Jurkat T lymphocytes (B) shown in Fig 2. Cells were transfected or electroporated with the lentiviral vector pfNL43-dE-EGFP encoding HIV-1, and treated for 24 h with 3 μM MG132 before lysis (at indicated times post-transfection/electroporation). The ubiquitylation profiles of untreated cells are also shown for comparison.

HIV-1–induced decrease in the abundance of SUMO-conjugated proteins raises a number of possibilities. As mentioned above, poly-SUMOylation can initiate substrate ubiquitination and proteasomal degradation. HIV-1 may induce proteasomal targeting and/or degradation of sumoylated host proteins, resulting in increased turnover. Alternatively, the virus may interfere with the conjugation/deconjugation pathway and antagonize SUMO modification of substrate proteins either by inhibiting the E1, E2, or E3 enzymes or by promoting SUMOs’ deconjugation from the modified substrates by enhancing the activities of SUMO-specific proteases (SENPs) (21). To distinguish between these possibilities, we first treated HIV-1–expressing HEK293 cells with MG132, a proteasome inhibitor. As shown in Fig 2A, pharmacologic blockade of the proteasomes did not considerably prevent the virus-induced loss of sumoylated proteins, suggesting that the latter does not reflect enhanced proteasomal turnover. Similarly, in CD4^+^ Jurkat T-cells, MG132 treatment did not significantly stabilize the cellular SUMO conjugates whose abundance still diminished upon HIV-1 expression (Fig 2B). To further verify proteasome inhibition, we followed the fate of ubiquitin conjugates by Western blot, which accumulated drastically in both cell lines in response to MG132 exposure (Fig S2A and B).

**Figure 3.**
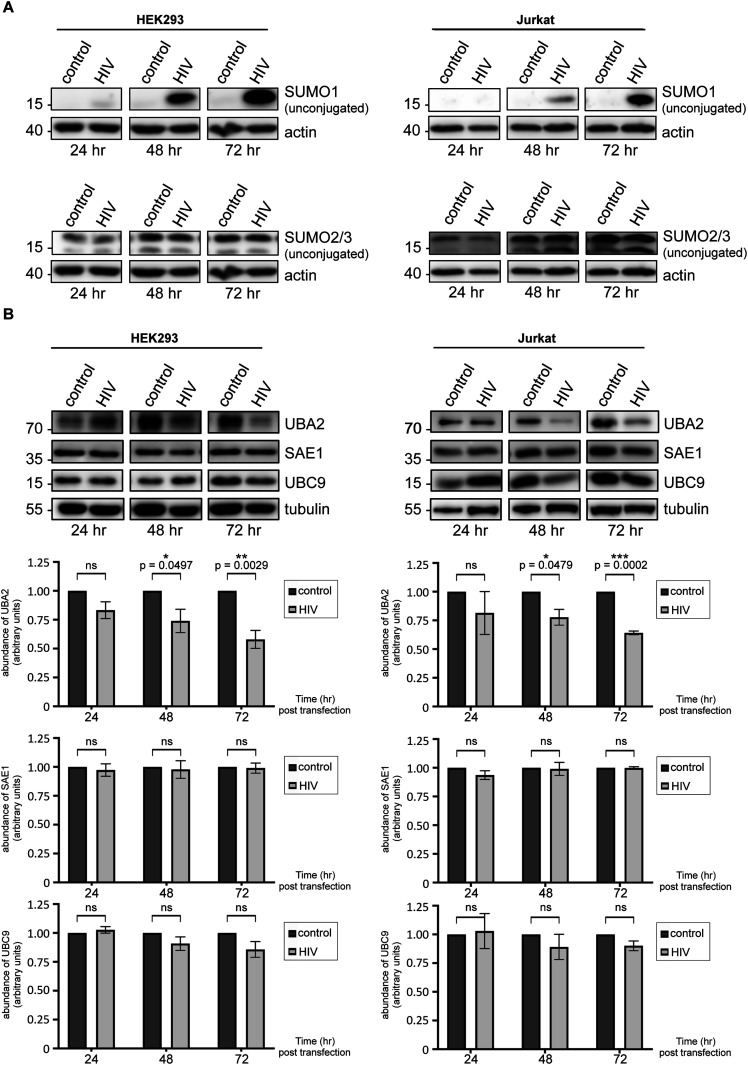
HIV-1 targets UBA2, a subunit of the SUMO E1–activating enzyme. **(A)** HEK293 cells or CD4^+^ Jurkat T lymphocytes were transfected or electroporated with the lentiviral vector pfNL43-dE-EGFP encoding HIV-1 as described above, followed by lysis at indicated times post-transfection to assess the level of free (unconjugated) SUMO1 and SUMO2/3 proteins by Western blot. **(B)** Western blots show UBA2, SAE1 and UBC9 protein levels in HEK293 and Jurkat cells. Cells were lysed and proteins were analyzed at indicated times after transfection or electroporation with HIV-1. HIV-1 specifically down-regulates the host UBA2 protein, a subunit of the heterodimeric SUMO E1 enzyme. Densitometric quantifications of UBA2, SAE1, and UBC9 signals were normalized to tubulin expression, which serves as a loading control. Data are presented as mean ± SEM (n ≥ 3 for HEK293 and Jurkat cells), asterisks denote statistical significance (*P*-values were calculated using *t* test assuming unequal variances, ns, not significant). Control, cells transfected with an empty vector backbone devoid of HIV-1 genes; HIV, cells expressing the HIV-1 genome.

Interestingly, the decline in global high molecular weight SUMO1 conjugates was accompanied by a time-dependent accumulation of the unconjugated SUMO1 peptide, implying that HIV-1 either prevents SUMO1’s conjugation to substrate proteins or promotes its deconjugation (Fig 3A). In line with the former possibility, after HIV-1 lentiviral expression in both HEK293 and Jurkat cells, we observed a sharp decrease in UBA2 protein levels, a subunit of the heterodimeric SUMO E1–activating enzyme (Fig 3B). Conversely, SAE1 (the other E1 subunit) and UBC9 (E2 conjugating enzyme) levels remained intact (Fig 3B). Similar results were also obtained in HeLa cells expressing the HIV-1 genome (Fig S1D and data not shown). Contrarily to SUMO1, we did not observe a significant accumulation of the unconjugated SUMO2/3 peptide after HIV-1 expression in HEK293 or Jurkat cells (Fig 3A). This observation likely reflects the well-documented differences in the natural abundance and conjugation behaviour of these two peptides. In cells, free SUMO1 is limiting in quantity, as most of this peptide is found attached to high-affinity substrate proteins, whereas free SUMO2/3 is abundant but remains mostly unconjugated unless cells are exposed to stress (21, 36). This difference in the conjugation behavior renders SUMO1 highly sensitive and responsive to changes in the enzymatic machinery, and could explain why UBA2 hampering by HIV-1 leads to a dramatic accumulation of this peptide, while not noticeably altering the abundance of free SUMO2/3. However, we cannot exclude the possibility that HIV-1 may also modulate SUMO1 gene expression, while not altering the expression of SUMO2/3.

**Figure S3.**
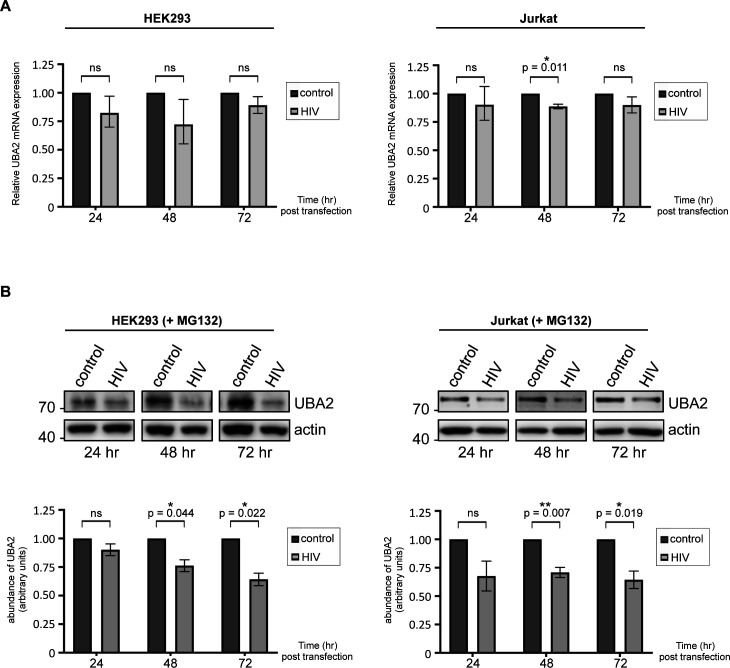
Further analysis of HIV-1–induced UBA2 loss. **(A)** Real-time PCR analyses of UBA2 mRNA levels in HEK293 cells or in CD4^+^ Jurkat T lymphocytes transfected or electroporated with the lentiviral vector pfNL43-dE-EGFP encoding the HIV-1 genome. Data are presented as mean ± SEM (n = 4 for HEK293 cells, n = 3 for Jurkat cells). *P*-values were calculated using *t* test assuming unequal variances. Control, cells transfected with an empty vector backbone devoid of HIV-1 genes; HIV, cells expressing the HIV-1 genome. Analyses were performed at indicated time points post-transfection/electroporation. **(B)** Western blot analysis of UBA2 protein in HIV-1–expressing HEK293 or Jurkat cells treated with the proteasome inhibitor drug MG132, as in Fig 2. Densitometric quantifications of UBA2 protein were normalized to actin expression, which serves as a loading control. Data are presented as mean ± SEM (n = 3), asterisks denote statistical significance (*P*-values were calculated using *t* test assuming unequal variances, ns, not significant). Control, cells transfected with an empty vector backbone devoid of HIV-1 genes; HIV, cells expressing the HIV-1 genome.

Subsequently, we attempted to understand the basis of the HIV-1–induced decline in UBA2 expression. To address this point, we first checked the UBA2 mRNA levels after transfection or electroporation of HIV-1 in HEK293 or Jurkat cells. Real-time PCR analyses did not indicate considerable changes in UBA2 transcript levels, suggesting that the virus did not impede the transcription of UBA2 gene (Fig S3A). To test whether HIV-1 enhances the degradation of UBA2 protein by the proteasomes, we used MG132 to inhibit the latter. Nevertheless, proteasomal blockage did not considerably stabilize UBA2 in HIV-1–expressing cells (Fig S3B), hinting at the existence of non-proteasomal proteolytic mechanisms that may consume this protein upon viral entry/expression.

Collectively, these data demonstrate that HIV-1 specifically targets the UBA2 subunit of the host SUMO E1 enzyme and induces its non-proteasomal degradation, thereby reducing the efficiency of global SUMO conjugation.

### Global leukocyte sumoylation is diminished independently of HIV-induced cytopenia during HIV disease

Because sumoylation is critical for immune function and for anti-viral defense, we investigated whether the sumoylation profile of the immune system is affected in vivo in HIV-1–infected individuals. Selective isolation of HIV-1–infected CD4^+^ T-cells from patients would be challenging, and we wanted to monitor a wider fraction of the immune system. Hence, we collectively analyzed the total leukocyte population, which includes PBMCs and granulocytes.

**Figure 4.**
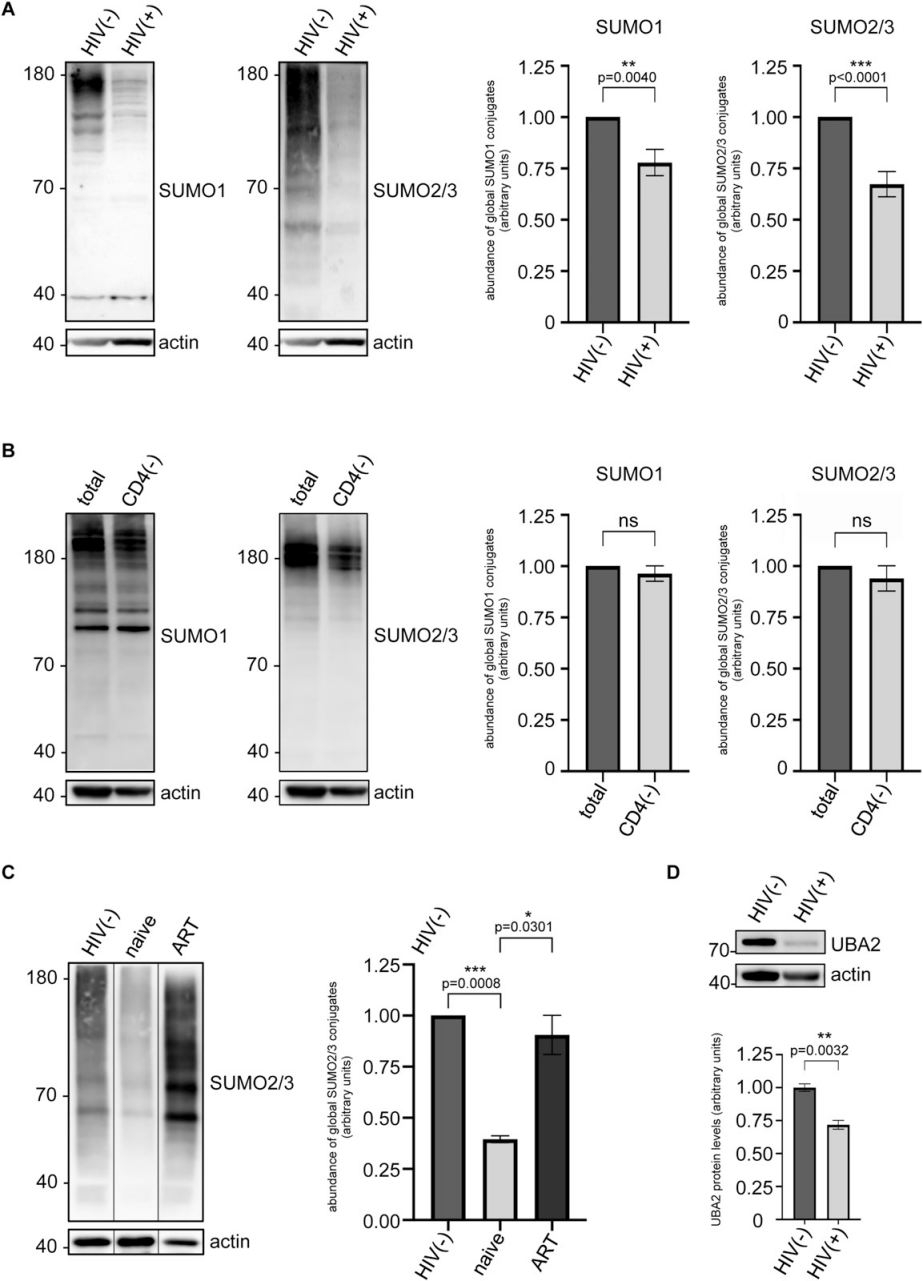
Global leukocyte sumoylation is impaired during HIV disease. **(A)** Western blots show SUMO conjugate levels in total leukocytes of a representative ART-naive HIV-1–infected individual (HIV(+)), in comparison with an uninfected individual (HIV(−)). Western blots were performed as described in Fig 1 and in the Materials and Methods section; sumoylated proteins were detected using human anti-SUMO1 and anti-SUMO2/3 antibodies. Actin: loading control (important note: equal amounts [20 μg] of protein were loaded in each well to allow fair comparison of SUMO profiles between HIV(−) and HIV(+) samples). Graphs (right panel) show densitometric quantifications of SUMO signals normalized to actin expression. Data are presented as mean ± SEM (n = 15 individuals for SUMO1, n = 19 individuals for SUMO2/3), *P*-values are indicated (using *t* test assuming unequal variances). Asterisks denote statistical significance. **(B)** The contribution of CD4^+^ cells to total leukocyte sumoylation was assessed by Western blot after ex vivo depletion of the former from the peripheral blood (leukocytes) of HIV-negative individuals. Total leukocytes (total: before depletion) and the CD4^+^ cell-deprived fraction (CD4(−)) are shown for comparison for a representative individual. Graphs show densitometric quantifications of SUMO signals normalized to actin expression. Data are presented as mean ± SEM (n = 4 individuals), *P*-values were calculated using *t* test assuming unequal variances, ns, not significant. Sumoylation in the CD4^+^ fraction is shown in Fig S4B. **(C)** Western blot shows leukocyte sumoylation profiles of a representative HIV-1–infected individual before (labeled as naive) and after anti-retroviral therapy (labeled as ART), with respect to that of an uninfected control individual (HIV(−)). Patient received ART for 3 mo after which the HIV-1 viral load became undetectable, yet the CD4^+^ count did not considerably rise. Graph shows densitometric quantifications of SUMO signals normalized to actin expression. Data are presented as mean ± SEM (n = 3 individuals), *P*-values are indicated (using *t* test assuming unequal variances). Asterisks denote statistical significance. **(D)** Western blot analysis of UBA2 in total leukocytes of a representative ART-naive HIV-1–infected individual (HIV(+)), in comparison with an uninfected individual (HIV(−)). 3 HIV(+) and 3 HIV(−) individuals were analyzed; graph shows densitometric quantifications of UBA2 signals normalized to actin expression (data are presented as mean ± SEM, n = 3 individuals, asterisks denote statistical significance; *P*-value is indicated, using *t* test assuming unequal variances).

**Figure S4.**
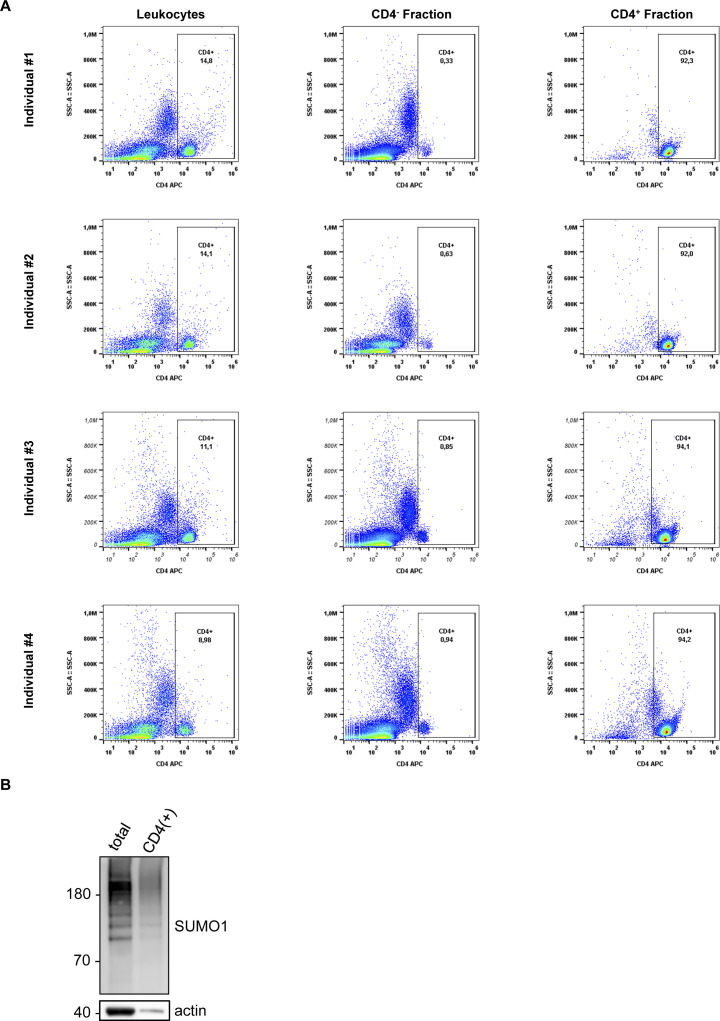
Further analysis of sumoylation in leukocytes. **(A)** CD4^+^ T-cell counts of the four HIV(−) individuals described in Fig 4B. Flow cytometry analyses indicate that CD4^+^ cells constitute about 8–15% of total leukocytes (PMBCs and granulocytes) of these individuals. Left, middle and right columns show CD4 positivity in total leukocytes, CD4^+^ cell-depleted (CD4(−)), and CD4^+^ cell populations, respectively. **(B)** Sumoylation in CD4^+^ cells, in comparison with that of total leukocytes. CD4^+^ cells were removed ex vivo from the peripheral blood (leukocytes) of an HIV(−) individual analyzed in Fig 4B. Total leukocytes (total: before depletion) and the CD4^+^ fraction (CD4(+)) are shown for comparison.

We collected peripheral blood samples from 19 ART (anti-retroviral therapy)-naive HIV-1–infected (HIV(+)) patients, as well as from 12 healthy donors belonging to an uninfected control group (HIV(−)). Patients belonged to a similar age-group with no known comorbidities (no diabetes, hypertension, cancer, thyroid, or cardiovascular disease) or co-infections (except patient #11 who tested positive for HBV: hepatitis B virus [Table 1]). Total leukocytes, including PBMCs and granulocytes, were isolated and subjected to protein extraction, followed by SDS–PAGE and immunoblotting with anti-SUMO1 or anti-SUMO2/3 antibodies to assess global sumoylation levels. In line with our in vitro findings, compared with the HIV(−) group, the HIV(+) group displayed on average a 22% (±0.064) decrease in global leukocyte sumoylation by SUMO1 (*P* = 0.004), along with a 33% (±0.061) decrease in sumoylation by SUMO2/3 (*P* < 0.0001) (Fig 4A). These findings suggest that HIV-1 may target global immune system sumoylation in vivo, possibly more severely affecting the SUMO2/3-conjugated proteins. Critically, equal amounts of total protein were loaded on gels when comparing HIV(−) and HIV(+) leukocyte profiles, suggesting that the reduction (or total loss in some cases) in global sumoylation in HIV(+) samples does not simply reflect CD4^+^ cell (or overall protein) depletion. To confirm this, we artificially depleted the CD4^+^ cells from the peripheral blood of HIV(−) individuals in vitro (Fig S4A). Western blot analysis of the remaining CD4^−^ fraction did not indicate a significant decrease in global SUMO conjugate levels (Figs 4B and S4B), implying that the contribution of CD4^+^ cells to overall leukocyte sumoylation is low. In addition, we studied three patients (patients #3, #4 and #12 in Table 1) who received ART during the course of this study, and on average, displayed a 60.5% (±0.014) decrease in global sumoylation by SUMO2/3 before the initiation of treatment (n = 3, *P* = 0.0008) (Fig 4C). Whereas all three patients achieved undetectable HIV-1 viral loads as early as 3 mo into the treatment, their CD4^+^ cell counts did not considerably increase (remained low between 80 and 250/mm^3^, a normal CD4^+^ count ranges from 500 to 1,200 cells/mm^3^); nevertheless, global leukocyte sumoylation levels were restored concomitant with this viral suppression (Fig 4C). As a consequence, these results suggest that the reduction in global leukocyte sumoylation is uncoupled from HIV-induced cytopenia and likely a direct effect of uncontrolled viral replication and HIV-1 interaction with the immune system.

**Table 1.** 19 HIV-1–infected and anti-retroviral therapy (ART)–naive individuals participated in this study.

Patient	CD4^+^ T count	HIV RNA (copy/ml)	Comorbidity and co-infections
1	0	62,519	None
2	0	281,611	None
3	13	506,119	None
4	20	909,805	None
5	23	1,448,862	None
6	28	3,862,507	None
7	30	365,300	None
8	32	570,578	None
9	39	317,400	None
10^a^	45	5,919	None
11^a^	62	1,538,498	Chronic Hepatitis B
12	65	14,190,707	None
13	91	744,873	None
14^a^	121	935,552	None
15	185	216,695	None
16	200	3,051,146	None
17	255	645,270	None
18^a^	343	214,343	None
19	350	2,717,724	None

a Only SUMO2/3 data were available for these four patients.

Table shows their CD4^+^ T-cell counts (in ascending order) and HIV-1 viral loads. Among these, 15 were analyzed for both SUMO1 and SUMO2/3; individuals #10, 11, 14, and 18 could be analyzed for SUMO2/3 only. Except for #11 who had chronic hepatitis B infection, none of the individuals had comorbidities (diabetes, hypertension, cancer, thyroid, or cardiovascular disease) or co-infections. Three of the patients (#3, #4 and #12) later received ART and were further analyzed in Fig 4C.

We then asked whether a potential decrease in UBA2 expression in vivo may contribute to the reduced global leukocyte sumoylation activity that we observe in HIV-1–infected individuals. To address this question, we used Western blot analyses to assess UBA2 protein levels in patients’ leukocytes. Intriguingly, in line with our findings in cell lines, HIV-1–infected individuals displayed a significant reduction in UBA2 expression (Fig 4D), suggesting that HIV-1 may target UBA2 also in vivo, among others.

All in all, our data indicate that during untreated HIV disease, global leukocyte sumoylation, in particular by SUMO2/3, is down-regulated in the infected patients in a therapy-reversible manner, unraveling another layer of complexity by which HIV-1 targets the host immune system in vivo.

## Discussion

SUMO peptides are critical regulators of innate immunity (21, 45, 52). SUMO is implicated in numerous signaling pathways that regulate immune function, including NF-κB, Toll-like receptor, and interferon signaling. For example, sumoylation finely tunes the processes that oversee interferon production during infection. Several interferon regulatory factors are sumoylated (52). In addition, SUMOs have recently emerged as crucial downstream mediators of interferon anti-viral activities, restricting pathogen replication (43). In general, SUMOs oppose the replication of viruses and bacteria. Overexpression of SUMO1 or SUMO2 in cultured HeLa cells significantly reduces infection by *L. monocytogenes* (42). Conversely, abrogation of sumoylation renders cells vulnerable to infections, for example, UBC9 depletion dramatically increases the efficiency of infection by HSV1 lacking its virulence factor ICP0 (46).

Recent landmark findings indicate that pathogens have evolved with strategies that modulate or impair host sumoylation, or attack sumoylated proteins, to evade host immune defenses (41, 53). As mentioned earlier, ICP0 selectively targets a subset of sumoylated proteins with known anti-viral functions and initiates their proteasomal degradation (46, 47, 49). Epstein–Barr virus encodes microRNAs that silence the expression of proteins, such as PML and RNF4, which play central roles in SUMO signaling (54, 55). Infection by the influenza virus also initiates wide-spread remodeling of the host sumoylation landscape (56). The avian adenovirus CELO encodes a protein called GAM1, which inactivates the SUMO E1 heterodimer and reduces the stabilities of the SUMO E1 and E2 (UBC9) enzymes, thereby efficiently down-regulating host cell sumoylation during infection (57). *L. monocytogenes* globally impairs host sumoylation by initiating non-proteasomal degradation of UBC9 (42). *Shigella flexneri* also abolishes sumoylation during infection by targeting the host UBC9 enzyme (58). A recent study revealed that *Klebsiella pneumoniae* reduces sumoylation to restrict host defense responses by either increasing the levels of SENP2, a SUMO-deconjugating enzyme, or by interfering with let-7 family of microRNAs that target the SUMO transcripts (43, 59). In this study, we provide the first evidence that HIV-1 is also capable of antagonizing cellular sumoylation, most likely via targeting of the host E1 enzyme, and further report that infection in patients is associated with a global demise in total leukocyte sumoylation.

Remarkably, the decrease in global sumoylation in leukocytes was slightly more pronounced for SUMO2/3 than it was for SUMO1 (33% versus 22%, respectively). Growing evidence indicates that protein modification by SUMO1 or SUMO2/3 may lead to different biochemical outcomes and regulate distinct physiological functions (21, 36). For example, in cells exposed to stress conditions such as oxidation and heat shock, SUMO2/3 is rapidly mobilized to be conjugated to thousands of target proteins as part of a major cellular response to cope with environmental assaults. In that respect, both SUMO2 and SUMO3 are essential for cells to survive through hyperthermic stress (37). Because HIV infection inflicts considerable damage and stress on cells, for instance by increasing reactive oxygen species levels (60, 61), the virus may try to cripple host anti-stress mechanisms by primarily disarming SUMO2/3 conjugation. Interestingly, chromatin occupancy by SUMO2/3 may finely tune viral latency or reactivation, or control the expression of host immune-related genes during infection by Kaposi’s sarcoma associated herpes virus (62, 63). Of note, we have seen in vitro in HEK293 and Jurkat cells that HIV-1 efficiently reduces sumoylation by both SUMO1 and SUMO2/3, implying that both paralogs may actually antagonize HIV-1 infection in vivo, though further studies are needed to explore this. However, we cannot rule out the possibility that primary and transformed cells may behave differently pertaining to the cellular pathways and mechanisms that are activated or silenced upon viral infection; for instance, paralog-specific suppression of sumoylation.

The virus-induced decline in cellular sumoylation may be explained by the dramatic loss of the UBA2 subunit of the host E1 enzyme. Viral factors targeting UBA2, or signals triggering its non-proteasomal degradation remain to be determined. HIV-1 expression did not cause a remarkable change in UBC9 levels, unlike previously reported for *L. monocytogenes* and *S. flexneri*, which also impair global protein sumoylation (41, 42, 58). Infections often stimulate proteasomal activity resulting in the increased turnover of sumoylated proteins, especially those that are conjugated with SUMO2/3. Indeed, immunoproteasomes are rapidly formed in response to many infections and display enhanced proteolytic activities (64, 65, 66, 67). Alternatively, one of the HIV-1 proteins may have a previously uncharacterized STUbL activity, or may interact with and activate a host STUbL to initiate proteasome-dependent degradation of sumoylated proteins. Importantly, the viral accessory proteins Vif, Vpx (found in HIV-2), and Vpu are known to counteract host restriction factors by serving as adaptors between these factors and certain ubiquitin E3 ligases and inducing their degradation (68). Nevertheless, pharmacologic inhibition of the proteasomes did not prevent or rescue HIV-1–induced down-regulation of host sumoylation, implying that defective conjugation rather than enhanced protein degradation is the most likely mechanism. This is confirmed by the reduction we observed in E1 (UBA2) levels. It remains to be determined whether HIV-1 also interacts with various SENPs to promote SUMOs’ deconjugation from protein substrates.

Because of the technical difficulty of isolating and studying patient-derived HIV-1–infected CD4^+^ cells, we decided to analyze total leukocyte sumoylation in vivo. Remarkably, we observed a significant decline in global leukocyte sumoylation in HIV(+) individuals. Importantly, we showed that the contribution of CD4^+^ cells, which constituted about 8–15% of all white blood cells (Fig S4A and reference 69), to total leukocyte sumoylation was low, if not negligible (Fig 4B). In addition, ART-induced viral suppression was sufficient to rescue leukocyte sumoylation in vivo, in a manner that was uncoupled from CD4^+^ cell recovery (Fig 4C). Therefore, we argue that the decrease in global leukocyte sumoylation that we observe in HIV-1–infected individuals does not simply reflect the depletion of CD4^+^ cells (cytopenia), but is most likely a direct outcome of uncontrolled viral replication and interaction with the host immune system components. Although more research is needed to fully understand the mechanisms leading to HIV-1–associated decline in global leukocyte sumoylation activity, our results suggest that UBA2 loss, at least in part, may contribute to this phenomenon in vivo (Fig 4D). HIV-1 is also known to interact with a subset of non-CD4^+^ cells (1). The overall effect of this non-canonical interaction may be more severe than previously anticipated, leading to significantly reduced levels of sumoylation in the entire leukocyte population. In addition, HIV-1–induced impairment of CD4^+^ cell function may disrupt signaling networks that regulate vital activities, such as sumoylation, in other immune cell types collectively (70, 71). Indeed, global cellular sumoylation is responsive to and controlled by major immune regulatory signals, such as those relayed by Toll-like receptors and interferon receptors, among others (43).

Other AIDS-defining opportunistic pathogens that resurface at low CD4^+^ counts (<200/mm^3^) may be potential confounders, possibly contributing to the sumoylation loss in vivo. For example, the CMV, which is known to target leukocytes, is a prominent opportunistic pathogen in advanced HIV disease (72, 73). Of note, only 1 of the 19 patients who participated in this study had a secondary infection other than HIV-1 (patient #11 who tested positive for HBV, Table 1), suggesting that HIV-1 is likely the primary culprit of sumoylation decrease in patients. Clinically successful ART reduces HIV-1 viral load to undetectable levels, thereby restoring immune function, and increases life expectancy. Remarkably, global leukocyte sumoylation was dramatically restored in ART-receiving patients, in a manner that preceded CD4^+^ cell recovery. The therapy-reversible nature of sumoylation loss in HIV disease is quite intriguing. Considering the critical roles of SUMOs in immune function, the recovery of sumoylation may actually facilitate immune restoration, including CD4^+^ cell recovery and expansion, in ART-receiving patients.

Interferon α displays limited anti-viral activity on HIV-1, weakly restricting its replication in vitro in cultured cells (43, 74). Interferon’s weak anti-HIV-1 activity depends on SUMO proteins and is blunted upon depletion of host SUMOs (43). Because SUMOs are crucial downstream mediators of interferon activity, viral targeting of host sumoylation may explain why interferons remain ineffective in fighting HIV-1 replication in vivo. In line with this hypothesis, although interferons also have a limited impact on HSV1 replication, they strongly restrict the replication of ΔICP0-HSV1, which is incapable of targeting host sumoylation (43, 75). Attacking the UBA2 subunit of the host E1 enzyme seems to be a very effective strategy for HIV-1 to dampen sumoylation. Deletion of HIV-1–encoded virulence factor(s) that attack UBA2 may sensitize the virus to interferon treatment. Importantly, in the clinic, targeting these putative factors by small molecules may be a viable treatment strategy as part of novel combination therapies that may also include interferon α.

HIV-1 disrupts or hijacks multiple cellular mechanisms and pathways upon infection, progressively damaging the immune system at the physiological level. We provide the first evidence that sumoylation is among the cellular processes that are targeted by HIV-1. Given the key functions of sumoylation in innate immunity and anti-viral defense, as well as in cell division, proliferation, and survival, disruption of this vital PTM during HIV-1 infection likely promotes viral replication, and also contributes to immune system deterioration in patients.

## Materials and Methods

### Cell culture, transfections, electroporation, and antibodies

HEK293 and HeLa cells were cultured in DMEM medium (Gibco) supplemented with 10% FCS, and transfected with the lentiviral vector (pfNL43-dE-EGFP) encoding the HIV-1 genome (purchased from Addgene) using the Effectene reagent (QIAGEN), according to manufacturer’s instructions. CD4^+^ Jurkat T lymphocytes were cultured in RPMI-1640 medium (Gibco), and electroporated with HIV-1 using the Neon Transfection System according to manufacturer’s instructions. The procedure was performed in Resuspension Buffer R, GFP-positivity was determined using BD Accuri C6 Flow Cytometer.

Human anti-SUMO1 (4930S) and anti-SUMO2/3 (4971S) antibodies were purchased from Cell Signaling Technology and human anti-actin (622102) antibody was purchased from BioLegend. Human anti-tubulin (sc-23948), human anti-GAPDH (sc-32233), human anti-UBA2 (sc-376305), human anti-SAE1 (sc-398080), anti-EGFP (sc-9996), anti–HIV-1-integrase (sc-69721) and anti–HIV-1-Rev (sc-69729) antibodies were purchased from Santa Cruz, and human anti-UBC9 (ab75854) antibody was from Abcam. Human anti-ubiquitin antibody was from R&D Systems (Clone FK2). All antibodies were used at 1:1,000 dilution for Western blot. The proteasome inhibitor drug MG132 was purchased from Sigma-Aldrich and used at 3 μM for 24 h before cell lysis.

### Real-time PCR analyses

The expression level of human UBA2 transcript was determined by quantitative PCR. RNA was isolated from HEK293 or Jurkat cells using Direct-Zol RNA Isolation Kit (Zymogen). cDNA synthesis was performed using SensiFAST cDNA Synthesis Kit (Bioline) according to manufacturer’s instructions. RT-PCR was performed using a SensiFAST SYBR No-ROX Kit (Bioline), as well as the following primers targeting UBA2 and GAPDH sequences: UBA2-sense 5′-CCC GAA AGC TAA TAT CGT TGC C-3′; UBA2-antisense 5′-ACT CGG TCA CAC CCT TTT TGA-3′; GAPDH-sense 5′-GGA GCG AGA TCC CTC CAA AAT-3′; GAPDH-antisense 5′-GGC TGT TGT CAT ACT TCT CAT GG-3′ (GAPDH was used as an internal control).

### Ex vivo depletion of CD4^+^ cells from the peripheral blood

To document the contribution of CD4^+^ cells to total leukocyte sumoylation, peripheral blood samples were collected from HIV-negative individuals. Isolation of mononuclear and polymorphonuclear leukocytes from patients’ peripheral blood was performed with density-gradient centrifugation using Lymphopure (#426201; BioLegend). After centrifugation, the buffy coat at interphase was collected without disturbing the other fractions and washed twice with PBS. CD4^+^ cells were depleted using magnetic separation with MS columns on a miniMACS Seperator (Miltenyi Biotec). Briefly, 2 × 10^6^ PBMCs were labeled with 40 μl human CD4 Magnetic MicroBeads (#130-045-101; Miltenyi Biotec) in 160 μl MACS buffer containing PBS (pH 7.2), 0.5% BSA and 2 mM EDTA. Samples were incubated at 4°C for 20 min and washed with 5 ml MACS buffer. The columns were equilibrated using 500 μl MACS buffer before cells were loaded onto the columns. Unlabeled (CD4^−^) cells were collected by washing the columns three times with 500 μl of MACS Buffer. For the collection of labeled cells (CD4^+^), the columns were removed from the separator and placed on a suitable collection tube and 1 ml of MACS Buffer was run through the columns.

For confirmation of CD4^+^ depletion, positive and negative fractions were stained with appropriate amounts of anti-human CD3 (#344806; BioLegend), anti-human CD4 (#317415; BioLegend), and anti-human CD8 (#345772; BD BioSciences) antibodies on ice for 30 min. The labeled cells were then washed with PBS and data acquisition was performed on an Accuri C6 (BD BioSciences) instrument. Data were analyzed with FlowJO v10.1 (BD Biosciences) software.

The remaining CD4^−^ fraction (that constituted about 85–92% of total leukocytes, Fig S4A) was subsequently lysed in standard Laemmli buffer containing β-mercaptoethanol and boiled at 95°C for 10 min. Lysates were cleared upon centrifugation at 11,000*g* for 10 min. SUMO conjugates were separated on 4–12% gradient gels (Invitrogen) or homemade 8% SDS–PAGE for further analysis by Western blot.

### Patients, leukocyte isolation, protein extraction, and analysis

Patients were selected from those that were admitted to Istanbul University, Cerrahpasa School of Medicine, Department of Clinical Microbiology and Infectious Diseases in Istanbul. Experiments were performed in accordance with the established institutional guidelines and approved protocols from the local ethics committee (Name: Istanbul University-Cerrahpasa, Ethics Committee for Clinical Research, Date and Number: 17 January 2017, 22142), and after obtaining informed consent from all participants. Patients’ characteristics are shown in Table 1. Isolation of mononuclear and polymorphonuclear leukocytes from patients’ peripheral blood was performed as described above. Isolated leukocytes were subsequently lysed in standard Laemmli buffer containing β-mercaptoethanol by boiling at 95°C for 10 min. Lysates were cleared upon centrifugation at 11,000*g* for 10 min. SUMO conjugates were separated on 4–12% gradient gels (Invitrogen) or homemade 8% SDS–PAGE for further analysis by Western blot. Importantly, equal amounts (20 μg) of total protein were loaded in each well to allow fair comparison of SUMO profiles between different samples, that is, HIV(−) or HIV(+) leukoctye extracts.

### Statistical analyses

Statistical analyses were performed using *t* test assuming unequal variances, *P*-values were calculated accordingly and indicated on all graphs. All data are presented as mean ± SEM (standard error of mean, n ≥ 3).

## Data Availability

The datasets generated in the current study are available from the corresponding author on reasonable request.

## Ethics Approval and Consent to Participate

Patients were selected from those that were admitted to Istanbul University, Cerrahpasa School of Medicine, Department of Clinical Microbiology and Infectious Diseases in Istanbul. Experiments were performed in accordance with the established institutional guidelines and approved protocols from the local ethics committee (Name: Istanbul University-Cerrahpasa, Ethics Committee for Clinical Research, Date and Number: 17 January 2017, 22142), and after obtaining informed consent from all participants in this study.

## Supplementary Material

Reviewer comments

## References

[1] Cohen MS, Shaw GM, McMichael AJ, Haynes BF (2011) Acute HIV-1 infection. N Engl J Med 364: 1943–1954. 10.1056/NEJMra1011874 21591946 PMC3771113

[2] Pope M, Haase AT (2003) Transmission, acute HIV-1 infection and the quest for strategies to prevent infection. Nat Med 9: 847–852. 10.1038/nm0703-847 12835704

[3] Fenwick C, Joo V, Jacquier P, Noto A, Banga R, Perreau M, Pantaleo G (2019) T-cell exhaustion in HIV infection. Immunol Rev 292: 149–163. 10.1111/imr.12823 31883174 PMC7003858

[4] Burgos J, Ribera E, Falcó V (2018) Antiretroviral therapy in advanced HIV disease: Which is the best regimen? AIDS Rev 20: 3–13. 10.24875/aidsrev.m17000010 29369304

[5] Goff SP (2007) Host factors exploited by retroviruses. Nat Rev Microbiol 5: 253–263. 10.1038/nrmicro1541 17325726

[6] Friedrich BM, Dziuba N, Li G, Endsley MA, Murray JL, Ferguson MR (2011) Host factors mediating HIV-1 replication. Virus Res 161: 101–114. 10.1016/j.virusres.2011.08.001 21871504

[7] König R, Zhou Y, Elleder D, Diamond TL, Bonamy GM, Irelan JT, Chiang CY, Tu BP, De Jesus PD, Lilley CE, (2008) Global analysis of host-pathogen interactions that regulate early-stage HIV-1 replication. Cell 135: 49–60. 10.1016/j.cell.2008.07.032 18854154 PMC2628946

[8] Brass AL, Dykxhoorn DM, Benita Y, Yan N, Engelman A, Xavier RJ, Lieberman J, Elledge SJ (2008) Identification of host proteins required for HIV infection through a functional genomic screen. Science 319: 921–926. 10.1126/science.1152725 18187620

[9] Chen L, Keppler OT, Schölz C (2018) Post-translational modification-based regulation of HIV replication. Front Microbiol 9: 2131. 10.3389/fmicb.2018.02131 30254620 PMC6141784

[10] Colomer-Lluch M, Castro-Gonzalez S, Serra-Moreno R (2020) Ubiquitination and SUMOylation in HIV infection: Friends and foes. Curr Issues Mol Biol 35: 159–194. 10.21775/cimb.035.159 31422939 PMC6987006

[11] Allen JD, Sanders RW, Doores KJ, Crispin M (2018) Harnessing post-translational modifications for next-generation HIV immunogens. Biochem Soc Trans 46: 691–698. 10.1042/BST20170394 29784645 PMC6013407

[12] Ribet D, Cossart P (2010) Pathogen-mediated posttranslational modifications: A re-emerging field. Cell 143: 694–702. 10.1016/j.cell.2010.11.019 21111231 PMC7112265

[13] Ji X, Gewurz H, Spear GT (2005) Mannose binding lectin (MBL) and HIV. Mol Immunol 42: 145–152. 10.1016/j.molimm.2004.06.015 15488604

[14] Fenouillet E, Jones IM (1995) The glycosylation of human immunodeficiency virus type 1 transmembrane glycoprotein (gp41) is important for the efficient intracellular transport of the envelope precursor gp160. J Gen Virol 76: 1509–1514. 10.1099/0022-1317-76-6-1509 7782780

[15] Pritchard LK, Harvey DJ, Bonomelli C, Crispin M, Doores KJ (2015) Cell- and protein-directed glycosylation of native cleaved HIV-1 envelope. J Virol 89: 8932–8944. 10.1128/JVI.01190-15 26085151 PMC4524065

[16] Harada S, Haneda E, Maekawa T, Morikawa Y, Funayama S, Nagata N, Ohtsuki K, Nagata N, Ohtsuki K (1999) Casein kinase II (CK-II)-mediated stimulation of HIV-1 reverse transcriptase activity and characterization of selective inhibitors in vitro. Biol Pharm Bull 22: 1122–1126. 10.1248/bpb.22.1122 10549869

[17] Idriss H, Kawa S, Damuni Z, Thompson EB, Wilson SH (1999) HIV-1 reverse transcriptase is phosphorylated in vitro and in a cellular system. Int J Biochem Cell Biol 31: 1443–1452. 10.1016/s1357-2725(99)00097-7 10641798

[18] Leng J, Ho HP, Buzon MJ, Pereyra F, Walker BD, Yu XG, Chang EJ, Lichterfeld M (2014) A cell-intrinsic inhibitor of HIV-1 reverse transcription in CD4(+) T cells from elite controllers. Cell Host Microbe 15: 717–728. 10.1016/j.chom.2014.05.011 24922574 PMC4072498

[19] Ohtsuki K, Maekawa T, Harada S, Karino A, Morikawa Y, Ito M (1998) Biochemical characterization of HIV-1 Rev as a potent activator of casein kinase II in vitro. FEBS Lett 428: 235–240. 10.1016/s0014-5793(98)00538-9 9654140

[20] Zheng Y, Yao X (2013) Posttranslational modifications of HIV-1 integrase by various cellular proteins during viral replication. Viruses 5: 1787–1801. 10.3390/v5071787 23863879 PMC3738961

[21] Celen AB, Sahin U (2020) Sumoylation on its 25th anniversary: Mechanisms, pathology, and emerging concepts. FEBS J 287: 3110–3140. 10.1111/febs.15319 32255256

[22] Gareau JR, Lima CD (2010) The SUMO pathway: Emerging mechanisms that shape specificity, conjugation and recognition. Nat Rev Mol Cell Biol 11: 861–871. 10.1038/nrm3011 21102611 PMC3079294

[23] Hay RT (2005) SUMO: A history of modification. Mol Cell 18: 1–12. 10.1016/j.molcel.2005.03.012 15808504

[24] Geiss-Friedlander R, Melchior F (2007) Concepts in sumoylation: A decade on. Nat Rev Mol Cell Biol 8: 947–956. 10.1038/nrm2293 18000527

[25] Wilkinson KA, Henley JM (2010) Mechanisms, regulation and consequences of protein SUMOylation. Biochem J 428: 133–145. 10.1042/BJ20100158 20462400 PMC3310159

[26] Seeler JS, Dejean A (2003) Nuclear and unclear functions of SUMO. Nat Rev Mol Cell Biol 4: 690–699. 10.1038/nrm1200 14506472

[27] Seeler JS, Dejean A (2017) SUMO and the robustness of cancer. Nat Rev Cancer 17: 184–197. 10.1038/nrc.2016.143 28134258

[28] Nacerddine K, Lehembre F, Bhaumik M, Artus J, Cohen-Tannoudji M, Babinet C, Pandolfi PP, Dejean A (2005) The SUMO pathway is essential for nuclear integrity and chromosome segregation in mice. Dev Cell 9: 769–779. 10.1016/j.devcel.2005.10.007 16326389

[29] Seufert W, Futcher B, Jentsch S (1995) Role of a ubiquitin-conjugating enzyme in degradation of S- and M-phase cyclins. Nature 373: 78–81. 10.1038/373078a0 7800043

[30] Dieckhoff P, Bolte M, Sancak Y, Braus GH, Irniger S (2004) Smt3/SUMO and Ubc9 are required for efficient APC/C-mediated proteolysis in budding yeast. Mol Microbiol 51: 1375–1387. 10.1046/j.1365-2958.2003.03910.x 14982631

[31] Tatham MH, Jaffray E, Vaughan OA, Desterro JM, Botting CH, Naismith JH, Hay RT (2001) Polymeric chains of SUMO-2 and SUMO-3 are conjugated to protein substrates by SAE1/SAE2 and Ubc9. J Biol Chem 276: 35368–35374. 10.1074/jbc.M104214200 11451954

[32] Matic I, van Hagen M, Schimmel J, Macek B, Ogg SC, Tatham MH, Hay RT, Lamond AI, Mann M, Vertegaal ACO (2008) In vivo identification of human small ubiquitin-like modifier polymerization sites by high accuracy mass spectrometry and an in vitro to in vivo strategy. Mol Cell Proteomics 7: 132–144. 10.1074/mcp.M700173-MCP200 17938407 PMC3840926

[33] Hendriks IA, D’Souza RC, Yang B, Verlaan-de Vries M, Mann M, Vertegaal AC (2014) Uncovering global SUMOylation signaling networks in a site-specific manner. Nat Struct Mol Biol 21: 927–936. 10.1038/nsmb.2890 25218447 PMC4259010

[34] Miteva M, Keusekotten K, Hofmann K, Praefcke GJ, Dohmen RJ (2010) Sumoylation as a signal for polyubiquitylation and proteasomal degradation. Subcell Biochem 54: 195–214. 10.1007/978-1-4419-6676-6_16 21222284

[35] Praefcke GJ, Hofmann K, Dohmen RJ (2012) SUMO playing tag with ubiquitin. Trends Biochem Sci 37: 23–31. 10.1016/j.tibs.2011.09.002 22018829

[36] Saitoh H, Hinchey J (2000) Functional heterogeneity of small ubiquitin-related protein modifiers SUMO-1 versus SUMO-2/3. J Biol Chem 275: 6252–6258. 10.1074/jbc.275.9.6252 10692421

[37] Golebiowski F, Matic I, Tatham MH, Cole C, Yin Y, Nakamura A, Cox J, Barton GJ, Mann M, Hay RT (2009) System-wide changes to SUMO modifications in response to heat shock. Sci Signal 2: ra24. 10.1126/scisignal.2000282 19471022

[38] Sahin U, Ferhi O, Jeanne M, Benhenda S, Berthier C, Jollivet F, Niwa-Kawakita M, Faklaris O, Setterblad N, de Thé H, (2014) Oxidative stress-induced assembly of PML nuclear bodies controls sumoylation of partner proteins. J Cell Biol 204: 931–945. 10.1083/jcb.201305148 24637324 PMC3998805

[39] Liu X, Wang Q, Chen W, Wang C (2013) Dynamic regulation of innate immunity by ubiquitin and ubiquitin-like proteins. Cytokine Growth Factor Rev 24: 559–570. 10.1016/j.cytogfr.2013.07.002 23953672

[40] Ribet D, Cossart P (2018) Ubiquitin, SUMO, and NEDD8: Key targets of bacterial pathogens. Trends Cell Biol 28: 926–940. 10.1016/j.tcb.2018.07.005 30107971 PMC7028394

[41] Ribet D, Cossart P (2010) SUMOylation and bacterial pathogens. Virulence 1: 532–534. 10.4161/viru.1.6.13449 21178495

[42] Ribet D, Hamon M, Gouin E, Nahori MA, Impens F, Neyret-Kahn H, Gevaert K, Vandekerckhove J, Dejean A, Cossart P (2010) Listeria monocytogenes impairs SUMOylation for efficient infection. Nature 464: 1192–1195. 10.1038/nature08963 20414307 PMC3627292

[43] Sahin U, Ferhi O, Carnec X, Zamborlini A, Peres L, Jollivet F, Vitaliano-Prunier A, de Thé H, Lallemand-Breitenbach V (2014) Interferon controls SUMO availability via the Lin28 and let-7 axis to impede virus replication. Nat Commun 5: 4187. 10.1038/ncomms5187 24942926

[44] Mabb AM, Miyamoto S (2007) SUMO and NF-kappaB ties. Cell Mol Life Sci 64: 1979–1996. 10.1007/s00018-007-7005-2 17530464 PMC11136396

[45] Adorisio S, Fierabracci A, Muscari I, Liberati AM, Ayroldi E, Migliorati G, Thuy TT, Riccardi C, Delfino DV (2017) SUMO proteins: Guardians of immune system. J Autoimmun 84: 21–28. 10.1016/j.jaut.2017.09.001 28919255

[46] Boutell C, Cuchet-Lourenço D, Vanni E, Orr A, Glass M, McFarlane S, Everett RD (2011) A viral ubiquitin ligase has substrate preferential SUMO targeted ubiquitin ligase activity that counteracts intrinsic antiviral defence. PLoS Pathog 7: e1002245. 10.1371/journal.ppat.1002245 21949651 PMC3174244

[47] Chelbi-Alix MK, de Thé H (1999) Herpes virus induced proteasome-dependent degradation of the nuclear bodies-associated PML and Sp100 proteins. Oncogene 18: 935–941. 10.1038/sj.onc.1202366 10023669

[48] Sloan E, Tatham MH, Groslambert M, Glass M, Orr A, Hay RT, Everett RD (2015) Analysis of the SUMO2 proteome during HSV-1 infection. PLoS Pathog 11: e1005059. 10.1371/journal.ppat.1005059 26200910 PMC4511656

[49] Boutell C, Sadis S, Everett RD (2002) Herpes simplex virus type 1 immediate-early protein ICP0 and is isolated RING finger domain act as ubiquitin E3 ligases in vitro. J Virol 76: 841–850. 10.1128/jvi.76.2.841-850.2002 11752173 PMC136846

[50] Zamborlini A, Coiffic A, Beauclair G, Delelis O, Paris J, Koh Y, Magne F, Giron ML, Tobaly-Tapiero J, Deprez E, (2011) Impairment of human immunodeficiency virus type-1 integrase SUMOylation correlates with an early replication defect. J Biol Chem 286: 21013–21022. 10.1074/jbc.M110.189274 21454548 PMC3121452

[51] Wimmer P, Schreiner S, Dobner T (2012) Human pathogens and the host cell SUMOylation system. J Virol 86: 642–654. 10.1128/JVI.06227-11 22072786 PMC3255802

[52] Hannoun Z, Maarifi G, Chelbi-Alix MK (2016) The implication of SUMO in intrinsic and innate immunity. Cytokine Growth Factor Rev 29: 3–16. 10.1016/j.cytogfr.2016.04.003 27157810

[53] Everett RD, Boutell C, Hale BG (2013) Interplay between viruses and host sumoylation pathways. Nat Rev Microbiol 11: 400–411. 10.1038/nrmicro3015 23624814

[54] Li J, Callegari S, Masucci MG (2017) The Epstein-Barr virus miR-BHRF1-1 targets RNF4 during productive infection to promote the accumulation of SUMO conjugates and the release of infectious virus. PLoS Pathog 13: e1006338. 10.1371/journal.ppat.1006338 28414785 PMC5413087

[55] Skalsky RL, Corcoran DL, Gottwein E, Frank CL, Kang D, Hafner M, Nusbaum JD, Feederle R, Delecluse HJ, Luftig MA, (2012) The viral and cellular microRNA targetome in lymphoblastoid cell lines. PLoS Pathog 8: e1002484. 10.1371/journal.ppat.1002484 22291592 PMC3266933

[56] Domingues P, Golebiowski F, Tatham MH, Lopes AM, Taggart A, Hay RT, Hale BG (2015) Global reprogramming of host SUMOylation during influenza virus infection. Cell Rep 13: 1467–1480. 10.1016/j.celrep.2015.10.001 26549460 PMC4660286

[57] Boggio R, Colombo R, Hay RT, Draetta GF, Chiocca S (2004) A mechanism for inhibiting the SUMO pathway. Mol Cell 16: 549–561. 10.1016/j.molcel.2004.11.007 15546615

[58] Sidik SM, Salsman J, Dellaire G, Rohde JR (2015) Shigella infection interferes with SUMOylation and increases PML-NB number. PLoS One 10: e0122585. 10.1371/journal.pone.0122585 25848798 PMC4388590

[59] Sa-Pessoa J, Przybyszewska K, Vasconcelos FN, Dumigan A, Frank CG, Hobley L, Bengoechea JA (2020) Klebsiella pneumoniae Reduces SUMOylation To Limit Host Defense Responses. mBio 11: e01733-20. 10.1128/mbio.01733-20 32994335 PMC7527722

[60] Ivanov AV, Valuev-Elliston VT, Ivanova ON, Kochetkov SN, Starodubova ES, Bartosch B, Isaguliants MG (2016) Oxidative stress during HIV infection: Mechanisms and consequences. Oxid Med Cell Longev 2016: 8910396. 10.1155/2016/8910396 27829986 PMC5088339

[61] Couret J, Chang TL (2016) Reactive oxygen species in HIV infection. EC Microbiol 3: 597–604. 28580453 PMC5450819

[62] Yang WS, Hsu HW, Campbell M, Cheng CY, Chang PC (2015) K-bZIP mediated SUMO-2/3 specific modification on the KSHV genome negatively regulates lytic gene expression and viral reactivation. PLoS Pathog 11: e1005051. 10.1371/journal.ppat.1005051 26197391 PMC4510548

[63] Chang PC, Cheng CY, Campbell M, Yang YC, Hsu HW, Chang TY, Chu CH, Lee YW, Hung CL, Lai SM, (2013) The chromatin modification by SUMO-2/3 but not SUMO-1 prevents the epigenetic activation of key immune-related genes during Kaposis sarcoma associated herpesvirus reactivation. BMC Genomics 14: 824. 10.1186/1471-2164-14-824 24267727 PMC4046822

[64] Kimura H, Caturegli P, Takahashi M, Suzuki K (2015) New insights into the function of the immunoproteasome in immune and nonimmune cells. J Immunol Res 2015: 541984. 10.1155/2015/541984 26636107 PMC4617869

[65] McCarthy MK, Weinberg JB (2015) The immunoproteasome and viral infection: A complex regulator of inflammation. Front Microbiol 6: 21. 10.3389/fmicb.2015.00021 25688236 PMC4310299

[66] Ferrington DA, Gregerson DS (2012) Immunoproteasomes: Structure, function, and antigen presentation. Prog Mol Biol Transl Sci 109: 75–112. 10.1016/B978-0-12-397863-9.00003-1 22727420 PMC4405001

[67] Murata S, Takahama Y, Kasahara M, Tanaka K (2018) The immunoproteasome and thymoproteasome: Functions, evolution and human disease. Nat Immunol 19: 923–931. 10.1038/s41590-018-0186-z 30104634

[68] Seissler T, Marquet R, Paillart JC (2017) Hijacking of the ubiquitin/proteasome pathway by the HIV auxiliary proteins, Viruses 9: 322. 10.3390/v9110322 PMC5707529 29088112

[69] Annacker O, Pimenta-Araujo R, Burlen-Defranoux O, Bandeira A (2001) On the ontogeny and physiology of regulatory T cells. Immunol Rev 182: 5–17. 10.1034/j.1600-065x.2001.1820101.x 11722620

[70] Meier-Schellersheim M, Varma R, Angermann BR (2019) Mechanistic models of cellular signaling, cytokine crosstalk, and cell-cell communication in immunology. Front Immunol 10: 2268. 10.3389/fimmu.2019.02268 31681261 PMC6798038

[71] Dustin ML, Chan AC (2000) Signaling takes shape in the immune system. Cell 103: 283–294. 10.1016/s0092-8674(00)00120-3 11057901

[72] Rice GP, Schrier RD, Oldstone MB (1984) Cytomegalovirus infects human lymphocytes and monocytes: Virus expression is restricted to immediate-early gene products. Proc Natl Acad Sci U S A 81: 6134–6138. 10.1073/pnas.81.19.6134 6091137 PMC391874

[73] Gerna G, Zipeto D, Percivalle E, Parea M, Revello MG, Maccario R, Peri G, Milanesi G (1992) Human cytomegalovirus infection of the major leukocyte subpopulations and evidence for initial viral replication in polymorphonuclear leukocytes from viremic patients. J Infect Dis 166: 1236–1244. 10.1093/infdis/166.6.1236 1331248

[74] Utay NS, Douek DC (2016) Interferons and HIV infection: The good, the bad, and the ugly. Pathog Immun 1: 107–116. 10.20411/pai.v1i1.125 27500281 PMC4972494

[75] Chee AV, Lopez P, Pandolfi PP, Roizman B (2003) Promyelocytic leukemia protein mediates interferon-based anti-herpes simplex virus 1 effects. J Virol 77: 7101–7105. 10.1128/jvi.77.12.7101-7105.2003 12768029 PMC156157

